# 651 Reduction in Fire-Related Admissions to a Large Regional Burn Center After Risk Reducing Interventions

**DOI:** 10.1093/jbcr/iraf019.280

**Published:** 2025-04-01

**Authors:** Dani Kruchevsky, Shaina Huda, Lorraine Todor, Mahmoud Hassouba, Xiangxia Liu, David Hill

**Affiliations:** University of Tennessee Health Science Center; University of Tennessee Health Science Center; UF Health Shands; University of Tennessee Health Science Center; University of Tennessee Health Science Center; Regional One Health

## Abstract

**Introduction:**

Residential fires account for most of the severe burn-related injuries and fatalities. Among established risk factors for burn injury include poverty and substandard housing characteristics. The burn center is located within a city ranked among the top 5 cities in overall poverty with populations greater than 500,000. Local municipality and fire department deployed several risk mitigation strategies, including education, installing residential smoke detectors, and demolishing abandoned urban buildings. The aim of our study was to evaluate the trend of fire-related admissions within our city.

**Methods:**

We conducted a retrospective analysis of burn admissions from July 2019 to June 2024. Assessed variables included demographics of the patients, date, location and circumstances of the incident, severity and distribution of the injury, presence of inhalation injury, CO poisoning, length of stay and surgical intervention. SAS 9.4 was used for data analysis. Descriptive statistics were used for demographic data, whereas chi-square test for the analysis of trends and risk factors.

**Results:**

During the 5-year period 1700 patients were admitted to the burn center, 481 of the burns occurred in our city, without significant variation between the years. Two hundred and four of the admitted patients suffered from fire-related injuries with 42 suffering inhalation injuries. The average age of the patients was 48.8 [14-97], 75.5% of the patients were male, 54% single and 37% had positive toxicology for substance abuse. Fourteen percent suffered an injury larger than 20% TBSA, 100 required surgical intervention, and 168 of the patients survived. Our data demonstrated a significant and consistent decrease in the number and ratio of patients admitted due to fire-related injuries (2019-49.5%, 2020- 58%, 2021- 38.9%, 2022- 38.8%, 2023 32.9%, P=0.004). Moreover, our data demonstrates significant distribution of the incidents to specific geographic location within the city.

**Conclusions:**

Fire related injuries cause significant morbidity and mortality, as well as major burden on the health system. Our data demonstrates significant and consistent reduction in admitted fire-related injuries since fire prevention intervention were initiated by the authorities. Moreover, recognized risk factors and geographical distribution of the incident’s locations will allow more efficient targeted interventions.

**Applicability of Research to Practice:**

Fire-related injuries cause significant morbidity and mortality, as well as major burden on the health system. The study is the first to demonstrate that risk reducing intervention resulted in significant and consistent reduction in admitted fire-related injuries in our geographic area. This result may encourage continued implementation of the risk reduction interventions and adoption of theses measures by other authorities.

**Funding for the Study:**

N/A

